# Reusing Jet Grouting Waste as Filler for Road Asphalt Mixtures of Base Layers

**DOI:** 10.3390/ma14123200

**Published:** 2021-06-10

**Authors:** Francesca Russo, Rosa Veropalumbo, Salvatore Antonio Biancardo, Cristina Oreto, Fabio Scherillo, Nunzio Viscione

**Affiliations:** 1 Department of Civil, Construction and Environmental Engineering, University of Naples Federico II, Via Claudio 21, 80125 Naples, Italy; francesca.russo2@unina.it (F.R.); salvatoreantonio.biancardo@unina.it (S.A.B.); cristina.oreto@unina.it (C.O.); nunzio.viscione@unina.it (N.V.); 2 Department of Chemical, Materials and Industrial Production Engineering, University of Naples Federico II, Piazzale Tecchio 80, 80125 Naples, Italy; fabio.scherillo@unina.it

**Keywords:** jet grouting waste, hot asphalt mixtures, cold recycled asphalt mixture, rheological properties of mastics, curing time effects, ITS, ITSM, resistance to permanent deformation

## Abstract

Secondary raw materials consist of production waste or material resulting from recycling processes, currently in large quantities, which can be injected back into the economic system as new raw materials. This study proposes jet grouting waste (JGW) as filler for hot and cold asphalt mixtures applied as base layers of road pavements and investigates the physical and mechanical properties. JGW is derived from soil consolidation performed during underground roadway tunnel construction. The research compares three asphalt mixtures: (a) hot mixture containing limestone aggregate-filler (HMA), (b) HMA containing JGW (HMAJ), (c) cold recycled asphalt mixture containing JGW (CRAJ). Leaching tests of JGW and reclaimed asphalt pavement (RAP) were conducted; the best configurations of the three mixtures were determined by using the volumetric method through gyratory compaction. Three mastics with filler-to-binder ratios reflecting those of the asphalt mixtures were investigated through delta ring and ball test and frequency sweep test at 0.05% stress by using a dynamic shear rheometer. The morphology of each mixture was further investigated by scanning electron microscopy. The results showed that CRAJ with 28 days of curing time reached the indirect tensile strength (ITS) of HMA (0.73 MPa) within 14 days and, among all studied mixtures, returned the lowest cumulative strain, which was on average 30% lower than that of HMA and HMAJ. The results of this study have shown that the cold alternative mixture, CRAJ, promotes the reuse of two types of waste, RAP and JGW, as it fully meets the reference Italian Technical Standard and ensures good mixture performance in addition to conserving natural resources.

## 1. Introduction

The 2030 Agenda for Sustainable Development envisages a world wherein every country enjoys sustained, inclusive, and sustainable economic growth [1]. Specifically, the ninth goal promotes resilient infrastructures that pursue sustainable management with the reduction of natural resources, replacing them with marginal materials.

Several studies on road construction have emphasized the mechanical and environmental benefits of hot asphalt mixtures (HMA) prepared by using waste and secondary materials [2], such as construction and demolition waste [3], stabilized bottom ashes from municipal waste incinerators [4], fly ashes from electricity facilities [5], waste foundry sand [6], and waste glass powder [7].

Skaf et al. [8] studied the effects of electric steelmaking slags (electric arc furnace (EAF) and ladle furnace steel slags (LF)) for preparing porous asphalt wearing layer without using limestone aggregates or fillers. Three types of mixtures were investigated: (a) a reference mixture of siliceous coarse aggregates and cement and 5% Pmb 45/80–60 by weight of the total mixture; (b) a mixture of siliceous coarse aggregates with 12.8% LF as fines and filler and 5% Pmb by weight of the total mixture; (c) a mixture containing 82.2% EAF slag as coarse aggregate and 13.6% LF slag as fines and filler and 4.2% Pmb by weight of the total mixture. The results showed that the last mixture had 11% higher indirect tensile strength (ITS) and helped in decreasing the permanent deformation by 7.6% when compared with the reference mixture of siliceous coarse aggregates and cement.

Dimter et al. [9] examined the physical and mechanical properties of asphalt mixtures for the base-wearing layers of a pavement with different contents of wood ash (WA) as the filler. Bitumen type B50/70 (4.9% by weight of the mixture) was used and four mixtures were prepared by using 0% (control), 25%, 50%, and 75% WA content. The Marshall stability (MS) and Marshall quotient (MQ) were found to increase when the WA content was increased up to 50%. In particular, MS increased by 4.5% (from 11.1 kN to 11.6 kN) and MQ increased by 15.0% (from 5.3 kN/mm to 6.1 kN/mm) when compared with the control mixture.

Sargin at al. [10] studied the effects of reusing rice husk ash (RHA) as filler for preparing a hot mixture for a binder layer containing 4.73% of 60–70 bitumen by weight of the aggregates. Four contents of RHA were investigated, namely, 25%, 50%, 75%, and 100% by weight of the limestone filler. The maximum MS value of 1563 kg was observed when 50% RHA was used, and the MS was 17% higher than the reference mixture containing only limestone filler.

Extensive efforts have been made to reuse reclaimed asphalt pavement (RAP), derived from the milling of old asphalt pavement layers [11], for making hot mixtures [12,13] and cold recycled mixtures [14,15,16], as no well-defined procedures exist.

Wang et al. [17] suggested a cold recycling procedure by using different additives: (1) recycling agent (an aromatic hydrocarbon, which is colorless and liquid at room temperature, representing approximately 0.5–1.0% of crude oil, with the capability to restore the aged binder), (2) emulsifying agent (lignin amine type slow-break cationic emulsifier obtained from the waste generated in the paper industry), (3) cement, and (4) modifier (acrylic copolymer emulsion). The researchers aimed to improve the engineering performance of an aged asphalt mixture with the maximum nominal size of 20 mm and bitumen–aggregate ratio of 4.3%. The results of the research showed that the highest value of fracture energy and elastic strain energy were achieved when 1.5% recycling agent, 0.1% emulsifying agent, and 0.05% modifier were added for preparing the cold mixture without cement.

Another issue that has been under investigation for several years concerns the effects of the curing process on the final mechanical properties of cold recycled asphalt mixtures [18].

Ferrotti et al. [19] investigated the properties of cold asphalt mixtures prepared by using 88% RAP, 10% river sand, and 2% mineral filler, with the addition of 4.5% and 4.0% bituminous emulsion (made up of 60% bitumen and 40% water) by the dry aggregate mass of binder and base layer solutions, respectively. The evolution of the indirect tensile stiffness modulus (ITSM) was studied at three different curing times ranging from 3 to 160 days: (1) oven-curing at 40 °C in the laboratory, (2) in the field, and (3) a curing time in the field followed by further oven-curing in the laboratory. The results showed that oven-curing at 40 °C may lead to an underestimation of the long-term stiffness of the mixture cured in the field, as the ITSM values obtained from the oven-curing at 40 °C were higher than the values of the field-cured material.

To date, as mentioned above, several waste and secondary materials have been investigated for preparing road asphalt mixtures; the reuse of jet grouting waste (JGW) derived from soil consolidation activities [20] has been gaining interest, although not for road pavement construction. Jet grouting technique is based on the injection of grout (water- and cement-based fluid mixture) into the soil at a very high flow rate (200–400 L/min), with a very high velocity of energy flow through small-diameter injection nozzles (1–10 mm) placed on a grout pipe or rod. The jet grout propagates radially with respect to the treatment axis from the borehole at a constant rate of rotation, separating the soil particles. The particles are then mixed and cemented with the jet grout. Then, the rod is slowly withdrawn toward the surface, forming a homogeneous mass of high-strength soil–cement body (the jet column) due to the solidification of the injected cement-based grout [21].

During soil consolidation work, the waste jet grout is expelled, together with the extracted soil that is replaced by the grout column; the mass solidifies on the ground surface and is called JGW. To date, the use of JGW is recognized in Italy, as laid down by the Italian Ministerial Decree (M.D.) of 5 February 1998, “Identification of non-hazardous waste subject to simplified recovery procedures under Articles 31 and 33 of Legislative Decree no. 22 of 5 February 1997”, for concrete grout and for foundation or sub-base of road embankments, after being subjected to environmental compatibility analysis.

Thus, the main objective of this paper is to evaluate the benefits derived from reusing JGW as filler for preparing hot asphalt mixtures (HMAJ) and cold recycled asphalt mixtures (CRAJ) for road pavement base layers using a traditional hot mix asphalt (HMA) as control mix.

**Figure 1:**
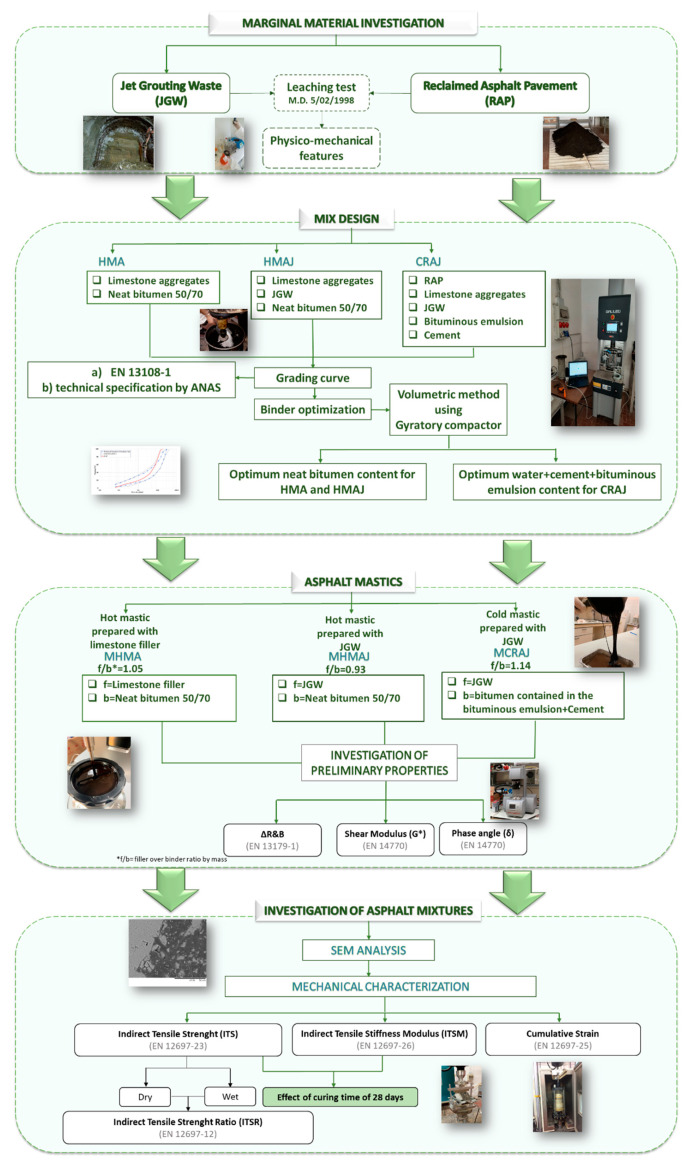
Flowchart of the analysis performed in this study.

Figure 1 depicts the workflow of the experimental research.

First, JGW and RAP were subjected to a leaching test to verify their compliance with the requirements of the Italian M.D. of 5 February 1998, and then, the mix design for the three asphalt solutions was conducted through gyratory compaction to maximize the JGW and RAP content in compliance with the mechanical requirement of local special tender specifications.

Subsequently, the following asphalt mastics employing the filler (f) to binder (b) (f/b) ratio used for the asphalt mixtures were prepared and investigated using delta Ring and Ball (ΔR&B) calculated between the asphalt mastics and neat 50/70 bitumen (EN 13179-1) and the frequency sweep test (FS) (EN 14770): (1) hot mastic prepared with only limestone filler (MHMA) with f/b ratio of 1.05, which meets the composition requirement of the HMA solution; (2) hot mastic prepared with JGW as filler (MHMAJ) with f/b ratio of 0.93, which meets the composition requirement of the HMAJ solution; and (3) cold mastic prepared with JGW as filler with f/b ratio of 1.14, which meets the composition requirement of the CRAJ solution, where the binder is made up of 10% cement and 90% bitumen emulsion (60% of neat 50/70 bitumen and 40% water).

Then, the asphalt mixtures were examined by using a scanning electron microscope (SEM), and the mechanical performance, such as ITS (EN 12697-23), moisture sensitivity (ITSR) (EN 12697-12), ITSM (EN 12697-26), and resistance to permanent deformation (EN 12697-25), were assessed. The effect of the curing time of the cold recycled asphalt mixture was examined by analyzing the variations in ITS and ITSM during 28 days.

In addition to the innovativeness of reusing JGW, this study contributes to the investigation of the benefits of adding two waste materials (JGW and RAP) for preparing cold recycled asphalt mixtures.

## 2. Materials and Methods

### 2.1. Limestone Aggregates

Coarse and fine limestone aggregates used for making hot and cold asphalt mixtures were from a quarry located in Southern Italy (Table 1 shows the main features).

### 2.2. Marginal Materials

#### 2.2.1. Jet Grouting Waste

**Figure 2:**
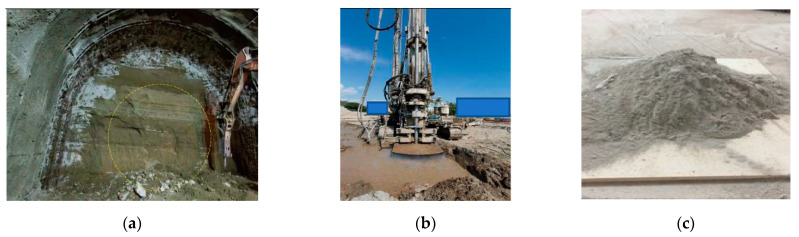
Jet grouting waste: (**a**) jet grouting columns, (**b**) JGW drawn to the surface; (**c**) JGW after grinding action.

The JGW (see Figure 2) was obtained from soil consolidation performed during the construction of an underground highway tunnel of an injection mixture composed of cement (500 ÷ 1100 kg for 1 m^3^) and water (650 ÷ 800 L for 1 m^3^), and it was reused as filler for preparing hot and cold asphalt mixtures. Before use, the JGW was subjected to a laboratory grinding process by means of a ball mill for nearly 2 h, after which no significant variation was found in the final grading size.

The main properties investigated for the JGW were density (EN 1097-6) and Rigden voids (EN 1097-4), which were determined to be 2.69 g/cm^3^ and 53%.

#### 2.2.2. Reclaimed Asphalt Pavement

**Figure 3:**
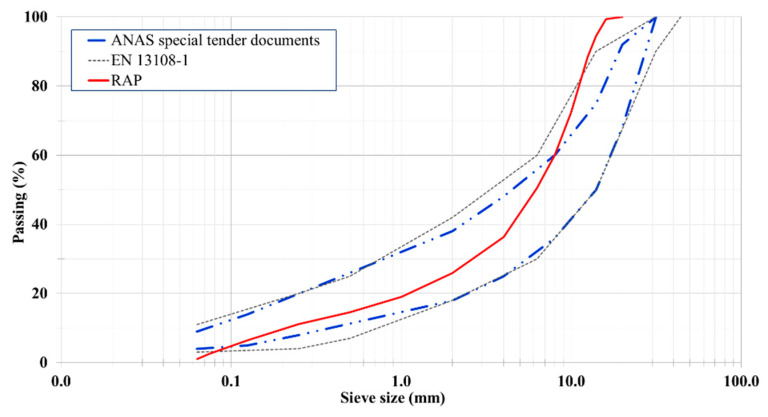
Grading curve of RAP according to standard requirements.

RAP was derived from the milling and stockpiling of old wearing courses and binder layers of road asphalt pavements. Table 2 shows its main properties, and Figure 3 shows its grading curve; RAP was used as black rock, and it needed the addition of limestone aggregates so that the composition met the standard.

#### 2.2.3. Leaching Test

**Figure 4:**
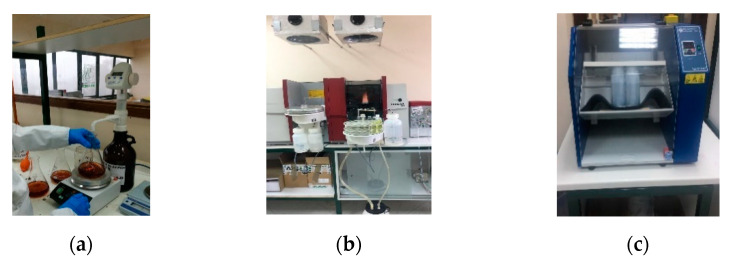
Leaching test: (**a**) COD titration, (**b**) APAT CNR IRSA 5130 Man. 29/03, and (**c**) agitation device.

The ground JGW and RAP were first subjected to the leaching test. The samples were prepared in compliance with UNI 10802:2013. Each sample was dried to minimize losses due to adhesion between the material and the equipment surface, after which, each sample was mixed and reduced to less than 50 g. The sample produced was then divided into two parts: (a) the first part was subjected to mineralization by using chemical agents assisted by a microwave source in accordance with EPA 3052 1996, and its chemical elements were evaluated, as shown in Table 3; (b) the second part was subjected to a leaching test conducted at 20 ± 5 °C according to EN 12457-2. The leaching test consisted of the following steps: (a) the metal elements were examined in the eluate by using plasma optical emission spectrometry according to the procedure described in EN ISO 11,885 (2009); (b) the composition of the dissolved solids was calculated in accordance with EN 15216; (c) the chemical oxygen demand (COD) was determined as a portion of the eluate by adopting the APAT CNR IRSA 5130 Man. 29/03 method (see Figure 4).

**Figure 5:**
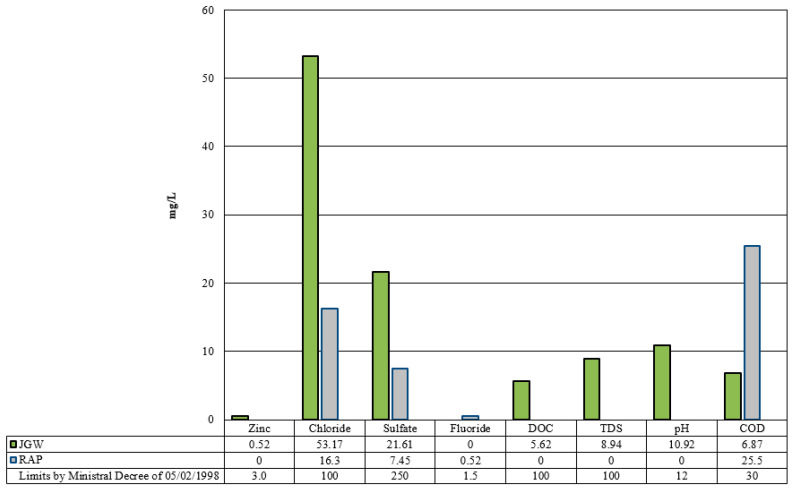
Leaching test results.

The leaching test results, shown in Figure 5, demonstrate that JGW and RAP do not exceed the environmental requirements fixed by the Italian M.D., mentioned above, and they may be used for preparing road asphalt mixtures by partial substitution of limestone aggregates.

### 2.3. Binders

A neat bitumen 50/70 penetration grade (B50/70) was used for preparing the hot solutions (HMA and HMAJ). By referring to the cold recycled asphalt mixture (CRAJ), portland cement 325R and an over-stabilized bituminous emulsion composed of 60% neat 50/70 bitumen and 40% water content were used to prepare the cold recycled asphalt mixture (CRAJ). Table 4 shows the main properties of the three binders.

### 2.4. Mix Design

#### 2.4.1. Grading Curve

**Figure 6:**
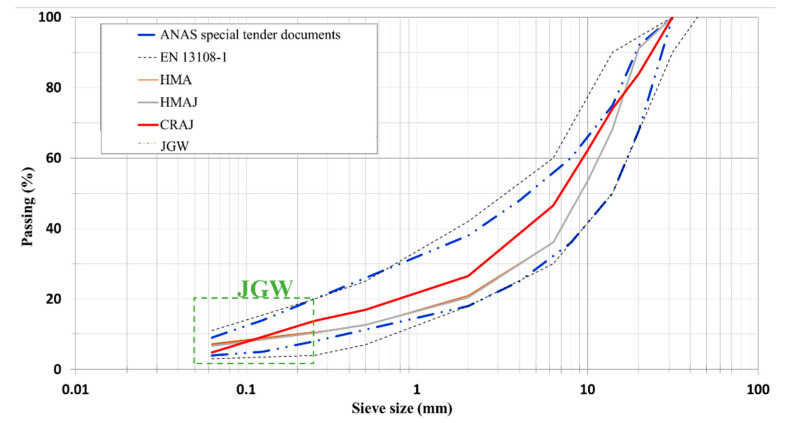
Grading curves for HMA, HMAJ, and CRAJ solutions vs. standard requirements.

First, the grading curves of the hot solutions, HMA and HMAJ, were obtained. The grading curves of HMAJ with 4% JGW filler and HMA with the limestone filler completely overlapped each other (see Figure 6). The specifications were as follows: (a) EN 13108-1 (see Table 1 in EN 13108—Overall limits of target composition—basic sieve set plus set 2) and (b) the technical specifications for HMA and HMAJ solutions for Southern Italy, provided by ANAS and shown in Table 5.

CRAJ has the following issues: (a) 4% JGW was used as filler in the previous hot solutions and (b) maximization of the RAP amount. The CRAJ mixture consisted of 76% RAP, 4% JGW as filler, and 20% limestone aggregates (see Table 5).

#### 2.4.2. Binder Optimization

To determine the optimum binder content (OBC) of the HMA and HMAJ solutions, a total of 24 cylindrical specimens were prepared at the compaction temperature of 160 °C under gyratory compaction energy (EN 12697-31) as required by ANAS special tender documents. The effects of five percentages of bitumen ranging from 3.5 to 5.0% by total weight of the aggregates with 0.25% increment were investigated. Cylindrical specimens with a diameter of 150 mm were compacted at N_max_ number of revolutions equal to 180, and the percentage of air voids (EN 12697-8) was verified against the desire value of 4%. The OBC for HMA was 3.8%, whereas that for HMAJ was 4.30%. The percentage increase by 0.5% of the OBC from HMA to HMAJ matched the Rigden void values: the percentage of Rigden voids in JGW was higher than that in the traditional limestone filler, which resulted in higher percentage of intergranular voids filled by bitumen in the HMAJ solution than in the HMA solution.

Next, we focused on the identification of the optimum content of water, cement, and bituminous emulsion of the CRAJ solution.

First, an optimum water + cement content was determined by preparing specimens with certain water content (ranging from 3% to 7% by total weight of the aggregates with an increment of 1%) and percentage cement content (ranging from 0.25% to 1% by total weight of the aggregates with increment of 0.25%) and by referring to literature [22,23]. Three specimens were prepared for each of the 20 water + cement combinations. Specifically, the limestone aggregates and JGW as filler were preheated at 105 °C and then added to the RAP, which was preheated at 60 °C; different preheating temperatures were selected because the materials had different moisture contents. The blend was then mixed in a planetary mixer for approximately 15 min and then compacted by using a gyratory compactor.

According to laboratory protocol, iterations for determining the optimum water + cement content were stopped at 180 gyrations when the cold mix asphalt reached a final bulk density close to that of HMA (2.52 g/cm^3^).

The first steps achieved a bulk density of 2.19 g/cm^3^ of CRAJ in the absence of the bituminous emulsion (bulk density by dimensions according to EN 12697-06_procedure D) with 5% water and 0.5% cement. As mentioned above, this value needs to be increased, as the bulk density value of the HMA solution investigated here equals 2.52 g/cm^3^ (bulk density using the saturated surface dry method according to EN 12697-06_procedure B).

The next phase focused on determining the optimum bituminous emulsion content by investigating the effects of four percentage contents (ranging from 3% to 6% by total weight of the aggregates with an increment of 1%) [24,25]. Three samples for each bituminous emulsion content were prepared by using a gyratory compactor, and these were added to the previously obtained specimens with optimal content of water + cement after preheating the bituminous emulsion content at 60 °C.

Finally, the most appropriate composition of CRAJ was determined as 0.5% cement, 5% water, and 5% over-stabilized bitumen emulsion, with bulk density of 2.52 g/cm^3^.

Before assessing the mechanical performance of the three optimized mixtures (HMA, HMAJ, and CRAJ), a preliminary study of the rheological properties of the mastics was performed to reveal the main correlations with the asphalt mixtures. The mastics were designed by following the f/b ratio used for designing each asphalt mixture, as described in Section 2.4.3.

#### 2.4.3. Asphalt Mastic Preparation

Three asphalt mastics were prepared [26], one for each of the three optimized mixtures, by using f/b ratios matching the corresponding weights observed in the optimized asphalt mixtures (HMA, HMAJ, CRAJ).

In particular, (a) the hot mastic made up of limestone filler (MHMA) met the specifications of the HMA solution with f/b ratio of 1.05 (the mixture contained 3.80% OBC and 4% limestone filler by total weight of the aggregates); (b) the hot mastic prepared with JGW as filler (MHMAJ) met the specifications of the HMAJ solution with f/b ratio of 0.93 (the mixture contained 4.30% OBC and 4% JGW as filler by total weight of the aggregates) (c) the cold mastic made up of JGW as filler met the specifications of the CRAJ solution with f/b ratio of 1.14 (the mixture contained 4% JGW filler, 0.5% cement, and 5% bitumen emulsion composed of 60% 50/70 neat bitumen as binder, by total weight of the aggregates).

**Figure 7:**
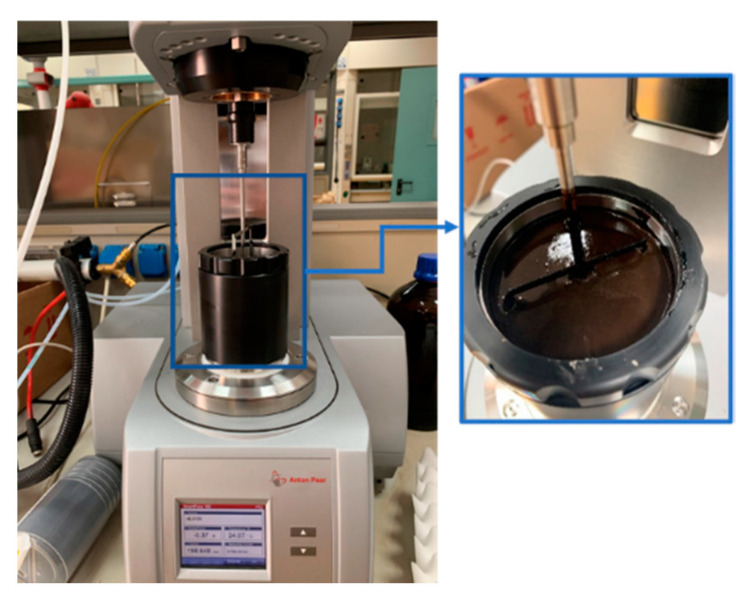
Building material cell for mixing asphalt mastics.

**Figure 8:**
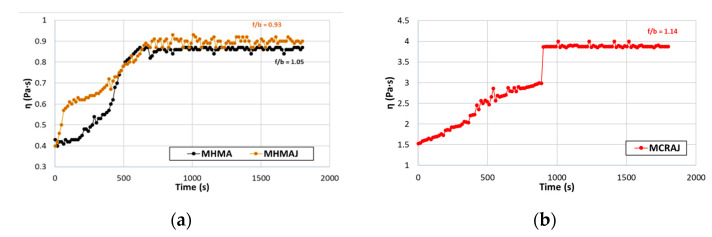
Viscosity variation by mixing time: (**a**) MHMA vs. MHMAJ and (**b**) MCRAJ.

A mechanical mixer equipped with an open blade impeller (see Figure 7) was used to stir the mastic, and the mixing temperature was kept constant (160 °C for the hot mastics, 60 °C for the cold mastics) to produce homogeneous blends with constant viscosity. The preheated filler was slowly added to the binder and each mastic was mixed continuously for thirty minutes, during which the viscosity (EN 13302) was examined. Figure 8 shows the viscosity diagrams against the mixing time.

Specifically, Figure 8b shows the viscosity variation by mixing time of the MCRAJ mastic solution, where it can be observed that the trend became horizontal after a sudden rise, at which point the bituminous emulsion broke.

**Figure 9:**
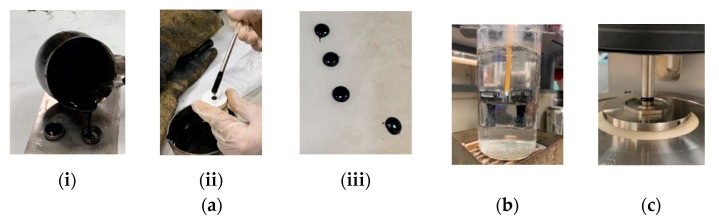
Investigation of asphalt mastics: (**a**) sample preparation: (**i**) R&B, (**ii**) FS, and (**iii**) final specimens for FS test; (**b**) Ring and ball test; (**c**) DSR configuration with 8 mm plate–plate geometry for conducting FS test.

It can be observed that all three mastics reached a constant viscosity with increasing mixing time; MHMA and MHMAJ reached the same constant viscosity (on average 0.89 Pa s) after nearly 10 min of mixing (see Figure 8a), whereas MCRAJ reached a constant viscosity after nearly 15 min, with the maximum value of approximately 3.8 Pa s. At the end of the blending phase, the mastics were poured into aluminum molds at room temperature for subsequent investigations (see Figure 9a).

### 2.5. Methods

This section deals with the assessment of the main-base rheological properties of the mastics prepared here and investigates the mechanical performance of the asphalt mixtures.

#### 2.5.1. Asphalt Mastics

ΔR&B temperatures (EN 13179-1) of the differences in softening point between the mastic and bitumen, between MHMAJ and MHMA, and between MCRAJ and MHMA were measured (see Figure 9b).

An FS test was then carried out by using a dynamic shear rheometer (DSR) (Anton Paar, Graz, Austria) by adopting the frequency values within the range from 0.1 to 10 Hz (20 observations were made with a gap of 0.1 for frequencies ranging from 0.1 to 1 Hz and a gap of 1 Hz for frequencies ranging from 1 to 10 Hz) at three test temperatures of 10, 25, and 40 °C, which were the same temperatures at which the ITSM values of the three asphalt mixtures (HMA, HMAJ, and CRAJ) were assessed; additionally, the permanent deformation of the three mixtures was assessed at the test temperature of 40 °C (see Section 2.5.2).

An “8 mm plate–plate geometry” with 2 mm gap (see Figure 9c) was adopted by applying 0.05% stress level identified through the strain sweep test (EN 14770) at 10 °C and under frequency of 10 Hz [27]. 

#### 2.5.2. Asphalt Mixtures

**Figure 10:**
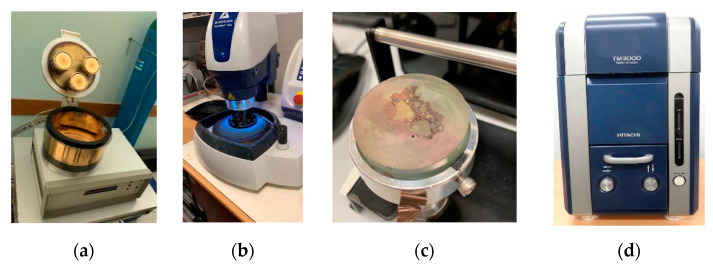
SEM specimen preparation: (**a**) smoothening of the specimen; (**b**) coating with the metal; (**c**) specimen type; and (**d**) Hitachi TM3000 device.

Firstly, the morphology of the three mixtures was investigated through SEM. Tiny portions of the three study mixtures were extracted and mounted in a cold epoxy resin. Subsequently, they were ground using a diamond disk with a grain size of 60 µm until a flat section appeared, revealing both the aggregate and bituminous mastic. Finally, the specimens were polished by using diamond disks with grain sizes of 10 µm and 5 µm. To make each of the three prepared specimens suitable for SEM analysis, they were coated with a thin gold layer using a sputter coater K650X (Quorum, East Sussex, United Kingdom) (see Figure 10). Each specimen was observed under a microscope TM3000 (Hitachi, Ito, Hirane).

**Figure 11:**
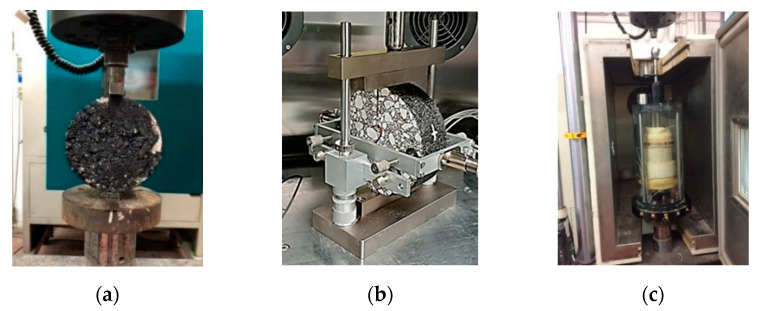
Asphalt mixture testing device: (**a**) ITS; (**b**) ITSM; (**c**) Cumulative strain.

The next step focused on the mechanical performance evaluation of the three mixtures. First, ITS assessment [28] under dry (EN 12697-23) and wet conditions (EN 12697-12) was carried out (see Figure 11a). In total, nine cylindrical specimens were prepared for each of the three study mixtures (HMA, HMAJ, and CRAJ); three were tested under dry conditions, and the remaining six under wet conditions. The specimens were prepared using gyratory compaction, as mentioned in the previous sections. The test specimens were brought to 25 °C in an air chamber and then stored for 4 h at the test temperature before testing (EN 12697-23). At the same time, to evaluate the ITS under wet conditions before exposing the specimens to the condition in the air chamber, six samples of each mixture were kept in a water bath at 40 °C for 72 h (EN 12697-12).

The ITS is obtained by using Equation (1).

(1) $$ITS=\frac{2\cdot P}{\pi \cdot d\cdot h}\;(\mathrm{MPa}),$$

where *P* is the peak load (N); *d* is the diameter of the specimen (mm); *h* is the height of the specimen (mm).

Furthermore, the variation in ITS with curing time was assessed. Laboratory curing was performed by placing each specimen in the oven for 3 days at 40 °C to attain accelerated-full curing conditions. Then, they were kept at 25 °C until a constant mass was observed [24,29]. The curing process was assessed by monitoring the ITS value over a period of 28 days (in particular at the 3rd, 5th, 10th, 14th, 20th, 25th, and 28th days), of which the first three days involved the accelerated curing mentioned above, and during the remaining period, the specimens were stored at room temperature before testing.

**Figure 12:**
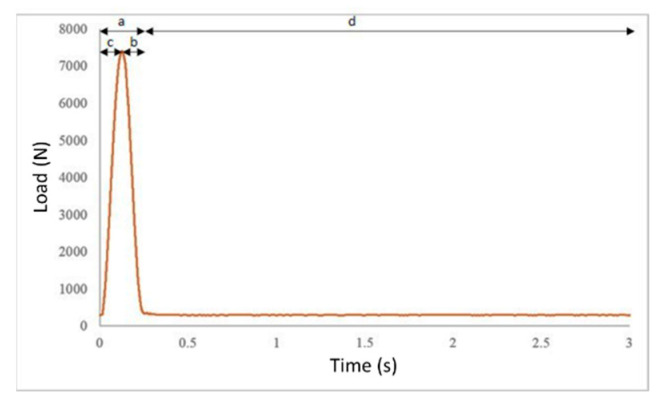
ITSM loading configuration.

Next, the ITSM was evaluated (see Figure 11b) by applying indirect tension to the cylindrical specimens (EN 12697-26—Annex C). The loading parameters and test configuration are shown, respectively, in Table 6 and Figure 12. The testing temperatures were 10 °C, 25 °C, and 40 °C. The applied load had a haversine waveform (see Figure 12) with a pulse load applied for 240 ms (see “part a” of the graph in Figure 12), corresponding to a rise time of 120 ms. The loading time consisted of two equal parts: rise time (see “part c” of the graph in Figure 12) and unloading time (see “part d” of the graph in Figure 12). Each pulse load was followed by an unloaded period (see “part b” of the graph in Figure 12). In total, five pulses were applied for the stiffness measurements. Using the measurements from the five load pulses, the ITSM (MPa) was determined using Equation (2).

(2) $$ITSM=\frac{F\cdot \left(\nu +0.27\right)}{\left(z\cdot h\right)}\;(\mathrm{MPa}),$$

where *F* is the peak value of the applied vertical load (N); *z* is the amplitude of the horizontal deformation obtained during the loading cycle (mm); h is the thickness of the specimen (60 mm); *ν* is Poisson’s ratio, equal to 0.35. As prescribed in EN 12697-26, all measurements were performed at a strain level of less than 50 micro-strains in order to ensure the linear viscoelastic zone.

In addition, the accelerated curing process was applied for the CRAJ specimens at 40 °C for 72 h, after which all specimens were stored for the remaining 25 days at room temperature of 25 °C. In particular, 21 specimens were prepared; sets of three specimens were randomly extracted after 3, 5, 10, 14, 20, 25, and 28 days and subjected to ITSM testing after conditioning for 4 h at each of the three test temperatures (10, 25, 40 °C).

Finally, the resistance to permanent deformation was evaluated for HMA, HMAJ, and CRAJ solutions by carrying out a cyclic compression test with confinement (EN 12697-25) (see Figure 11c).

Two cylindrical test specimens were prepared for each mixture. The diameter of the specimen was 100 mm, and the height was 75 mm in compliance with EN 12697-25. The test was performed at 40 °C. The specimens for the CRAJ solution were conditioned beforehand for 72 h at the test temperature [18]. A haversinusoidal pressure of $\sigma_{a}\left(t\right)$ (see Equation (3)) was applied for 10,000 loading cycles according to the standard.

(3) $$\sigma_{C}+\sigma_{a}\left(t\right)=\sigma_{C}+\sigma_{V}\cdot (1+sin\left(2\pi \cdot f\cdot t\right)),$$

where *σ_c_* is the confining stress (kPa); *σ_a_(t)* is the cyclic pressure as a function of time (kPa); *σ_v_* is the amplitude of the haversinusoidal pressure (kPa); *f* is the frequency (Hz); *t* is the time (s).

The results are expressed in terms of the cumulative axial strain, *ε_n_*, calculated by using Equation (4).

(4) $$\varepsilon_{n}=100\frac{h_{0}-h_{n}}{h_{0}}\;\;(\%),$$

where *h*_0_ is the height of the specimen after preloading (mm) and *h_n_* is the height of the specimen after *n* load applications equal to 10,000 (mm).

## 3. Results 

### 3.1. SEM Characterization

**Figure 13:**
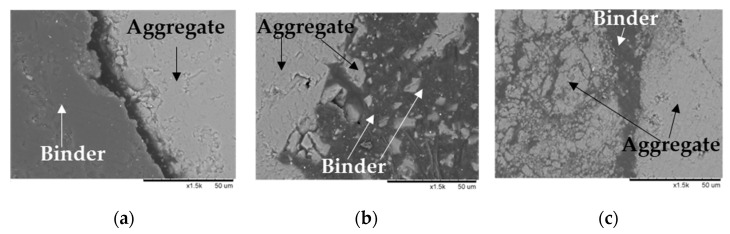
SEM characterization at 1500× magnification: (**a**) HMA, (**b**) HMAJ, and (**c**) CRAJ.

Figure 13a shows the morphology assessment of the HMA solution, where a surface break between the binder and aggregates of nearly 10 µm can be observed, probably born of high surface stress that caused binder shrinkage and detachment of the binder from the aggregates.

Figure 13b shows the morphology of the HMAJ solution. It can be observed that the binder shows better encapsulation of the aggregates than that observed in HMA.

Figure 13c shows the morphology of the CRAJ solution on the 28th curing day; the results show how the binder (cement plus bituminous emulsion) wraps more homogeneously around the aggregates as a solid skeleton and fills more voids than in the previous cases, also showing a greater thickness of the binder around the aggregates. Figure 13b,c show the geometric irregularity in the surface texture and non-spherical shape of the aggregate-filler particles of HMAJ and CRAJ solutions. The rough surface of JGW filler and RAP, than the traditional limestone aggregate, offers a good internal friction and interlocking. This condition favors an effective filler–binder bond; as a result the binder appears much more homogeneously distributed on the aggregate surface and well absorbed by the filler and the aggregate, affecting the final stiffness of the mixture

### 3.2. Properties of Asphalt Mastics

The addition of JGW for preparing hot (MHMAJ) and cold mastics (MCRAJ) resulted in a more general increase in the softening point when compared with a) the neat bitumen and b) the mastic prepared with only traditional limestone filler and bitumen (MHMA). 

The ΔR&B value between MHMAJ and the neat 50/70 bitumen was 67.80 °C, whereas that between MHMA and neat 50/70 bitumen was 26.20 °C. The difference in softening point between MHMAJ and MHMA was 41.6 °C (the softening point increased from 75 °C for MHMA to 116.6 °C for MHMAJ), emphasizing that MHMAJ was stiffer than the traditional MHMA, despite its f/b ratio being lower (0.93) than that for MHMA (1.05).

The MCRAJ solution was stiffer than the neat bitumen (with ΔR&B value equal to 81.40 °C), as well as the other two asphalt mastic solutions (MHMA and MHMAJ); The ΔR&B value between MCRAJ and MHMA was +55.20 °C, whereas that between MCRAJ and MHMAJ was +13.6 °C.

**Figure 14:**
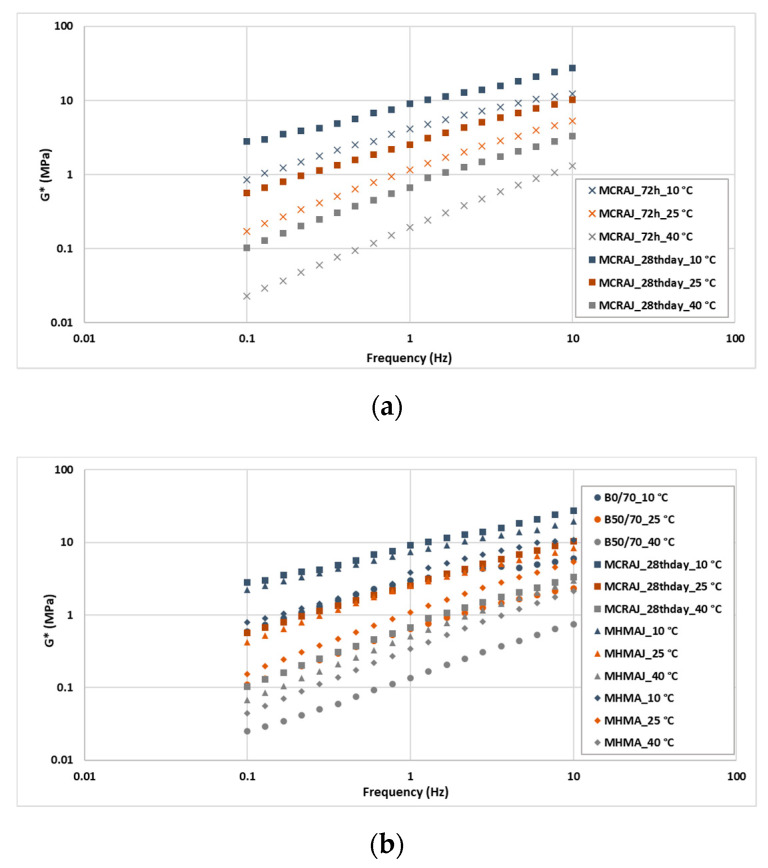
Isothermal plot of shear modulus for comparison between (**a**) CMRAJ by curing time and (**b**) neat bitumen vs. asphalt mastics.

**Figure 15:**
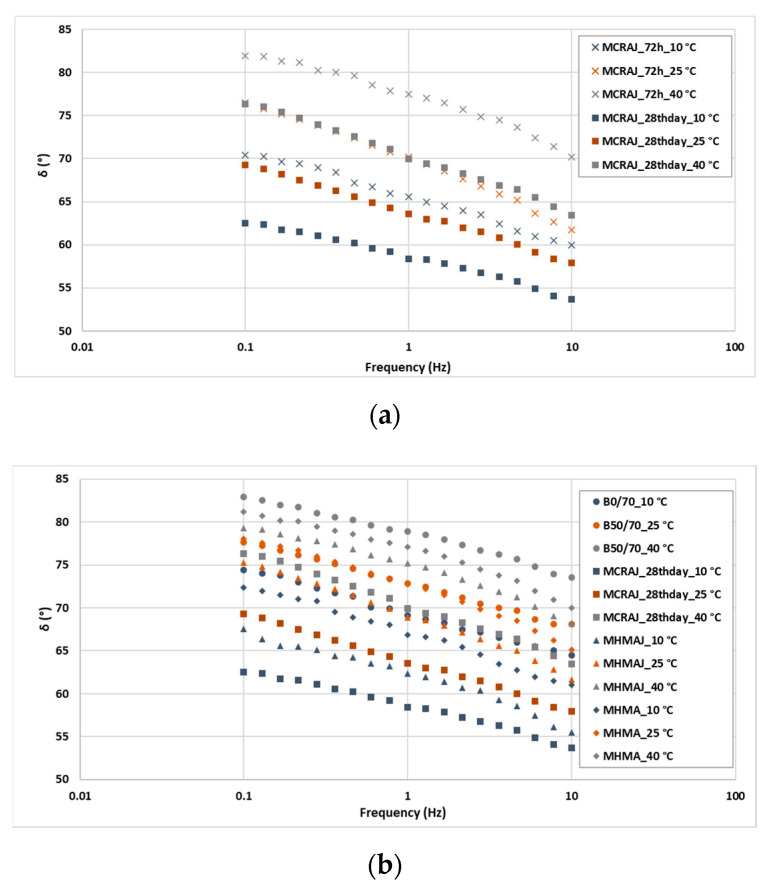
Isothermal plot of phase angle for comparison between (**a**) CMRAJ by curing time and (**b**) neat bitumen vs. asphalt mastics.

To comprehensively understand the effect of the stiffness provided by each filler, the asphalt mastics were analyzed through the FS test by assessing the values of G* and phase angle (δ) at 10, 25, and 40 °C by changing the frequency. The isothermal was plotted with G* or the phase angle on the *x*-axis and frequency ranging from 0.1 to 10 Hz on the *y*-axis (see Figure 14 and Figure 15).

An initial analysis was conducted on the cold mastic. It was decided to investigate the effect of the curing time (a) after the accelerated curing process at 40 °C for 72 h and (b) on the 28th curing day at room temperature, i.e., 25 days after the accelerated curing. The results in Figure 14a show that the shear modulus on the 28th curing day was higher than that calculated after the accelerated curing process. In particular, the results of G* were 113, 111, and 197% higher at the test temperatures of 10, 25, and 40 °C, respectively. Furthermore, from Figure 15a, it can be observed that on the 28th curing day, the elasticity of the mastic increased at all test temperatures (10, 25, and 40 °C), resulting in 10% lower phase angle values on average than those of MCRAJ after the accelerated curing process. 

By comparing between B50/70, the hot asphalt mastics (MHMA and MHAMJ), and the cold mastic on the 28th curing day, it is possible to observe in Figure 14b that all the mastics are stiffer than B50/70. The lowest increase in stiffness, an average of 54%, was recorded for MHMA at 10 °C, and the highest increase (an average of 369%) was observed for MCRAJ at 40 °C on the 28th curing day. The asphalt mastic solution containing JGW showed higher G* than MHMA for all frequencies investigated in this study. Particularly, MHMAJ and MCRAJ had an average of 109, 101, and 60% higher G* than MHMA at 10, 25, and 40 °C, respectively.

Between the two mastics containing JGW, MCRAJ on the 28th curing day was the stiffest. In fact, the values of G* were 29, 16, and 23% higher, respectively, than those of MHMAJ at 10, 25, and 40 °C.

Both the asphalt mastics containing JGW had lower phase angles than the B50/70 and MHMA asphalt solutions (see Figure 15b). The δ values of MCRAJ on the 28th curing day and MHAJ were on average 5% lower for all test temperatures investigated in this study, whereas they were on average 10, 8, and 5% lower than those of MHMA at 10, 25, and 40 °C, respectively. MCRAJ was more elastic than MHMAJ on the 28th curing day, because the phase angle of MCRAJ was on average 6% lower than that of MHMAJ.

From these results, it can be stated that the addition of a marginal filler (JGW) in both hot and cold blended mastics allows stiffer mastics than traditional mastics.

### 3.3. Asphalt Mixture Performance

#### 3.3.1. ITS Measurements

First, the effect of curing time on the ITS of the CRAJ solution was assessed.

**Figure 16:**
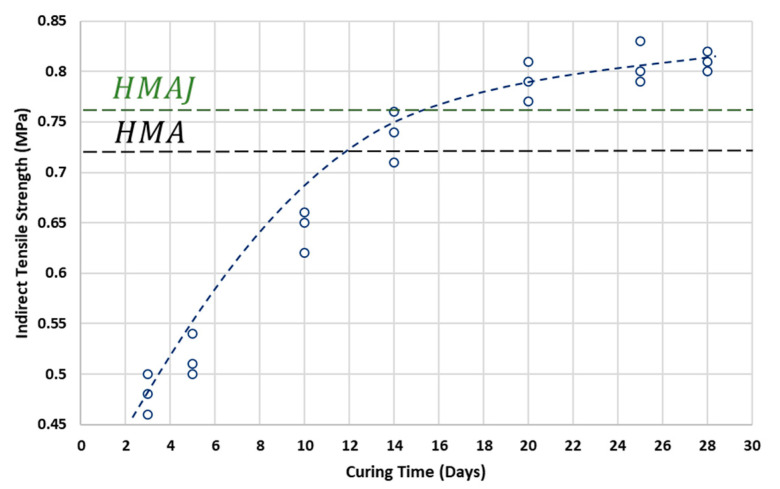
Dry ITS evolution of CRAJ solution with curing time.

Figure 16 shows the mean dry ITS values of CRAJ as a function of the number of days of curing time. It can be observed that CRAJ attains the ITS of HMA (the mean ITS value of the HMA solution is 0.72 MPa) just before 14 days of curing time under dry conditions, but it attains the ITS of HMAJ (the mean dry ITS value of the HMAJ solution is 0.76) before 20 days. On reaching 28 days of curing time, CRAJ attains the highest ITS value of 0.81 MPa.

It should be noted that the increase in ITS of the CRAJ mixture does not increase constantly with the curing interval. In fact, from 5 to 10 days and from 10 to 14 days, the ITS increased, on average, by 21% and approximately 5%, respectively. The increase in ITS gradation was greater in the first stage (5–10 days) than in the remaining period of the curing time, as the cohesive behavior of the mixture was more consolidated in the last step of the process.

**Figure 17:**
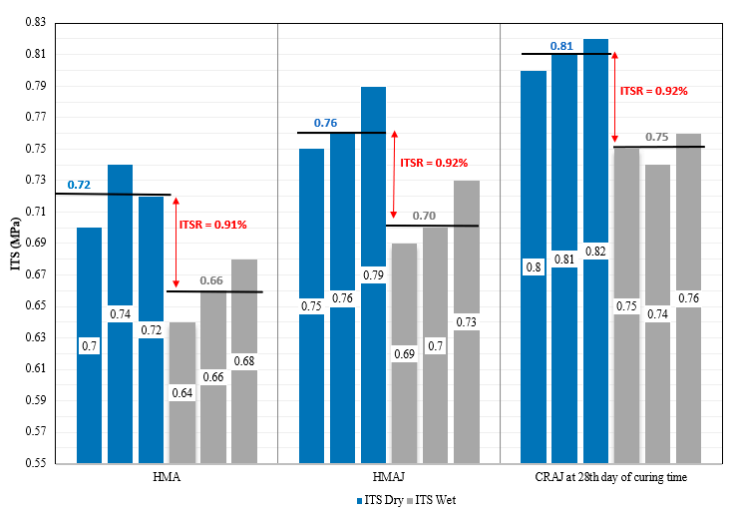
Dry vs. wet ITS.

Figure 17 shows the ITS results for all the mixtures (HMA, HMAJ, and CRAJ), tested under dry and wet conditions.

First, it may be observed that the mean ITS of HMAJ (0.76 MPa) is 5.5% higher than that observed for the traditional HMA (0.72 MPa) under dry conditions, and 6% higher under wet conditions (the mean value of wet ITS for HMAJ is 0.70 MPa; the mean value for HMA is 0.66 MPa).

The results of the CRAJ mixture correspond to the 28th day of curing time. The CRAJ mixture exhibited higher mean ITS resistance (0.81 MPa under dry conditions and 0.75 MPa under wet conditions) than the other mixtures; specifically, a higher mean ITS value was observed than the traditional HMA (mean increase of 12.5% under dry conditions and 13.6% under wet conditions) and HMAJ (mean increase of 6.5% under dry conditions and 7.1% under wet conditions).

The results showed low water sensitivity of the bituminous specimens because the indirect tensile strength ratio was higher than 90% (the lower limit imposed by ANAS); specifically, the best performance was achieved by adding JGW to the mixtures in the hot design and for CRAJ on the 28th day of curing time.

#### 3.3.2. ITSM

**Figure 18:**
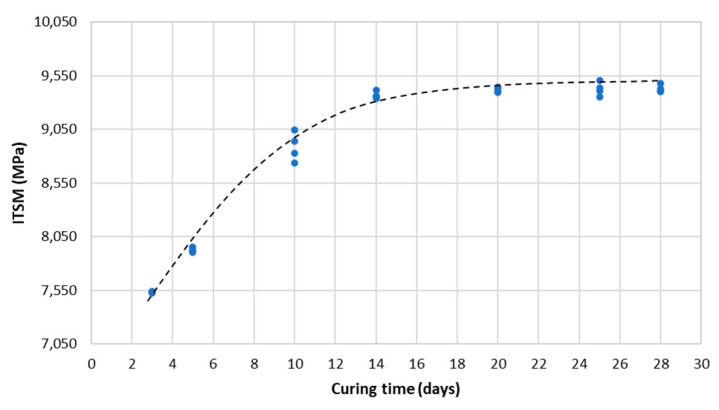
ITSM of CRAJ mixture at 25 °C vs. curing time.

Figure 18 shows an example of the evolution of ITSM at 25 °C of the CRAJ specimens vs. the curing time. It possible to observe that the stiffness increased up to the 14th day but remained almost constant between 14 and 28 days, reaching a maximum value of 9420 MPa on the 28th day of curing. This was because all curing time phenomena are strictly related to water evaporation, which reduces the water volume and causes a sharp increase in the air void volume during the first phase, as well as emulsion breaking and cement hydration, which are connected to environmental conditions [16].

The ITSM of the hot asphalt mixtures (HMA and HMAJ; see Table 7) were assessed as the mean value for the four specimens at the test temperatures of 10 °C, 25 °C, and 40 °C. As mentioned earlier, the ITSM values for the CRAJ solutions correspond to the 28th day of curing time.

Table 7 shows that the ITSM values follow a linear trend, increasing, on average, by almost 46% from 40 °C to 25 °C and from 25 °C to 10 °C, for all mixtures. The values of all solutions are in line with the limits imposed by the technical reference standard in Italy [24] (3500 MPa at 40 °C, 4000 MPa at 25 °C, and 5000 MPa at 10 °C). At each temperature, the CRAJ solution shows higher ITSM values than the HMA and HMAJ solutions (5.75, 18, and 33.3% higher on average at 10, 25, and 40 °C, respectively).

#### 3.3.3. Cumulative Strain

**Figure 19:**
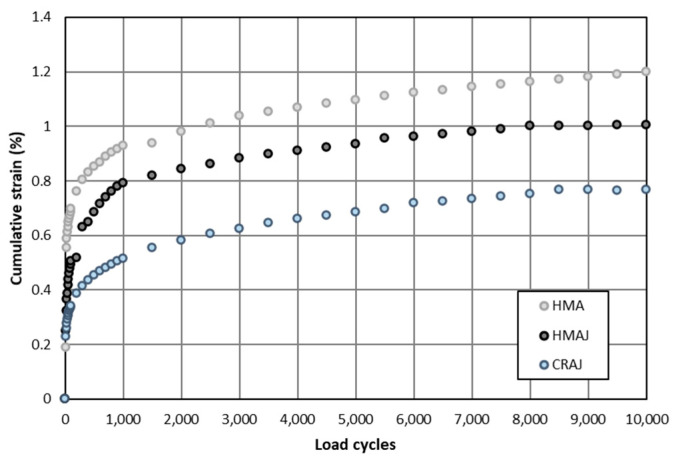
Cumulative strain vs. load cycles.

The results of cumulative strain obtained in the triaxial compression test are shown in Figure 19, where it is evident that the CRAJ solution analyzed after 28 days of curing time has the lowest cumulative strain value (on average, 30% lower than that of HMA and HMAJ). By comparing the alternative mixtures containing JGW as filler with HMA, it can be observed that the cumulative strain is reduced from 41% at the beginning of the load cycle to 16% at the end of the load cycle for HMAJ and from 53% to 35% for CMRAJ. Both reach a constant cumulative strain at around 9000 cycles, unlike HMA, which continues to deform even at the end of the loading cycles. These results demonstrate the contribution of JGW in reducing the permanent deformations of the mixtures, when added at both hot and cold mixing temperatures.

## 4. Discussion

This study aimed to investigate the reuse of JGW as marginal filler in hot asphalt mixtures and cold recycled asphalt mixtures. After investigating the environmental (leaching test in compliance with the M.D. 5 February 1998) and physical properties of JGW and RAP, the results of which established the feasibility of reusing JGW, the mixtures were subjected to a volumetric test.

The hot asphalt mixture contains 4% JGW (HMAJ) and requires 0.5% more OBC than HMA. Regarding the optimization phase of the cold recycled asphalt mixture with JGW (CRAJ), a mixture with bulk density close to that observed for the traditional hot mix (2.52 g/cm^3^) and with the following composition: 76% RAP, 4% JGW, and 20% limestone aggregates with 5% bituminous emulsion, 5% water, and only 0.5% cement content by weight of the aggregates was used. The value of the optimized cement content is lower than that suggested in literature [29], which is approximately 1.5% on average, due to the addition of JGW.

Next, the base properties of the asphalt mastics were investigated by preparing one mastic for each mixture by following the f/b ratio adopted for the mix design of each solution (HMA, HMAJ, CRAJ). Specifically, the effect of curing time was investigated for MCRAJ; the stiffness of the mastics evaluated on the 28th curing day at room temperature was higher than that calculated after the accelerated curing process for 72 h at 40 °C. Both mastics containing JGW showed the best performance when compared with the mastic containing the traditional limestone filler. These results are reflected in the higher ITS and ITSM values (on average, 9.7 and 19.2%, respectively) of the mixtures containing JGW at all three test temperatures investigated in this study when compared with the traditional mixture (HMA). In particular, the cold mixture was found to have better resistance to deformation than the remaining mixtures, with cumulative strain lower by 33% and 20% when compared with that of HMA and HMAJ.

To compare all parameters investigated for all mastics and mixtures, normalization was carried out by using Equation (5), where the minimum value represents the best performance (δ (at 10, 25, and 40 °C) and ε_n_), whereas in Equation (6), the highest value is the best performance (G* (at 10, 25, and 40 °C), ITS_wet,_ ITS_dry,_ ITSR, ITSM (at 10, 25 and 40 °C)).

(5) $$n_{ij}=1-\frac{a_{i\_k,j}-min\;a_{i\_k,j}}{max\;a_{i\_k,j}-min\;a_{i\_k,j}}\;,$$

(6) $$n_{ij}=1+\frac{a_{i\_k,j}-max\;a_{i\_k,j}}{max\;a_{i\_k,j}-min\;a_{i\_k,j}}\;,$$

where

- $a_{i\_k,j}$ is the i-th value assumed by the k-th parameter (G* (at 10, 25, and 40 °C), **δ** (at 10, 25, and 40 °C), ITS_wet_, ITS_dry_, ITSR, ITSM (at 10, 25 and 40 °C), and *ε_n_*, a total of 13 parameters) for the j-th asphalt solution (mastic or mixture).
- $min\;a_{i\_k,j}$ is the minimum value *a* obtained from all i-th values measured for each k-th parameter related to each j-th asphalt blend.
- $max\;a_{i\_k,j}$ is the maximum value *a* obtained from all i-th values measured for each k-th parameter related to each j-th mastic and mixture.

**Figure 20:**
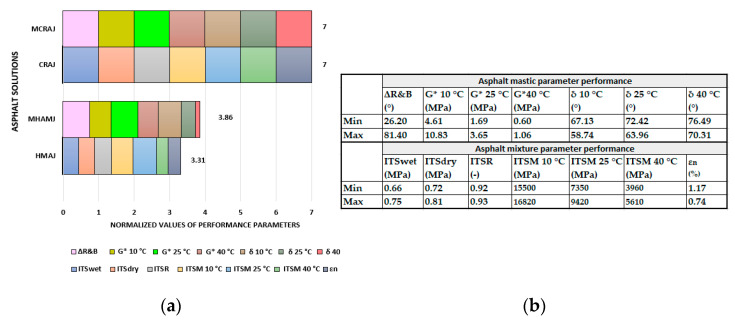
Comparison of solutions by normalizing the performance parameters: (**a**) performance diagram and (**b**) max and min values for each parameter performance.

Figure 20 shows the normalized value of each mechanical performance indicator and the sum of all values normalized for each mastic and mixture, which emphasize the overall high performance of each solution. The cold recycled mixture and mastic show an overall high performance for all indicators (seven variables were investigated through laboratory test) for a total of 7, on the contrary of HMAJ, that equals 3.31. The mastics exhibited almost the same trend.

## 5. Conclusions

The use of marginal materials as substitutes for natural materials plays an important and strategic role in both hot and cold mix design processes of road asphalt pavements, because they help to reduce (a) the use of virgin resources involved in the process without negative effect on the final mechanical performance of the mixtures and (b) negative environmental effects associated with the activities related to mining materials (aggregates and bitumen, mostly) and subsequent laying and maintenance activities. 

This study aimed to promote the technical feasibility of reusing jet grouting waste (JGW) as a substitute for traditional limestone filler in hot mix asphalt (HMAJ) or when it is combined with RAP for preparing CRAJ.

Overall, the following conclusions can be drawn:

- JGW as a filler helps to increase the ΔR&B temperature between the MHMAJ mastic (f/b ratio of 0.93) and neat 50/70 bitumen, as well as that between the MHMAJ mastic and MHMA mastic (f/b ratio of 1.05). MHMAJ has a lower ΔR&B than the cold mastic solution (MCRAJ), but the stiffening power of the JGW filler makes it possible to reach a higher ΔR&B when compared with traditional hot bituminous mastic made of limestone filler and neat bitumen.
- It was found that the accelerated curing process for 72 h at 40 °C was not satisfactory for evaluating the performance of the cold mastic in terms of G*. It is necessary to cure the mastics for 28 days to attain better performance than the hot mastics prepared with traditional limestone filler and JGW.
- The SEM analysis showed a good homogenous microstructure of the HMAJ and CRAJ solutions when compared with the traditional HMA.
- The curing time analysis of the cold asphalt mixture revealed that the CRAJ, investigated over 28 days, attained the ITS value of HMA before 14 days and matched the highest ITS observed for HMAJ in 20 days.

In conclusion, the cold alternative mixture (CRAJ) promotes the reuse of two types of waste, as it fully meets the reference Italian Technical Standard and ensures good mixture performance in addition to conserving natural resources. It should be noted that the suggested solutions should be adopted after performing a proper analysis of the costs and environmental effects of the processes. This is one of the future goals of this research. In addition, the correlations between the rheological properties of the mastics and different mix designs of the CRAJ solution will be investigated by changing the bituminous emulsion and/or cement type, the RAP (derived from other layers and not just from the base layer as in this case study), and/or the supply location of the JGW, and the life cycle requirements will be assessed.

## Figures and Tables

**Table 1:** Main properties of the limestone aggregates and filler.

Aggregate Size	Density	Los Angeles	Shape Index	Flattening Index	Equivalent Sand	Rigden Voids
	(g/cm^3^)	(%)	(%)	(%)	(%)	(%)
	EN 1097-6	EN 1097-2	EN 933-4	EN 933-3	EN 933-8	EN 1097-4
Coarse Aggregates						
31.5–16 mm	2.68	–	4	16	–	–
10–16 mm	2.69	16	4	8	–	–
6–12 mm	2.71	16.4	8	11	–	–
Fine Aggregates						
Sand	2.71	–	–	–	80	–
Filler	2.73	–	–	–	–	46

**Table 2:** Main features of RAP.

Parameters	Unit	Value	Standard
Size designation	–	20 RA 0/16	EN 13108-8
Foreign matter content	%	1.5	EN 12697-42
Bulk density	g/cm^3^	2.52	EN 1097-6
Water absorption	%	1.6	EN 1097-6
Sand equivalent	%	71	EN 933-8
Flakiness index	%	10	EN 933-3

**Table 3:** Chemical compositions of JGW and RAP.

Parameter	Concentration in mg/kg	
	JGW	RAP
Antimony	0.2	0.7
Arsenic	15.2	0.6
Beryllium	–	1.4
Cadmium	0.11	8.5
Calcium	70,350	–
Cobalt	10.5	14.1
Chromium (total)	21.1	36.4
Iron	13300	185
Magnesium	4750	–
Manganese	4.5	–
Nickel	11.5	25.1
Lead	0.8	4.5
Silicon	185,150	–
Copper (total)	19.5	11.6
Tin	4.4	2.8
Vanadium	4.59	3.1
Zinc	70.5	15.7

**Table 4:** Binder properties.

Parameters	Unit	Value	Standard
Bitumen			
Penetration @ 25 °C	dmm	68	EN 1426
Softening point	°C	48.8	EN 1427
Dynamic viscosity @ 150 °C	Pa s	0.25	EN 13702
Bituminous Emulsion			
Water content	%	40	EN 1428
pH value	–	4.2	EN 12850
Settling tendency at 7 days	%	5.8	EN 12847
Cement			
Initial setting time	min	112	EN 196-3
Compressive strength at 2 days	MPa	27.8	EN 196-1
Compressive strength at 28 days		61.2	
Volume constancy	mm	0.52	EN 196-3

**Table 5:** Mixture composition and their volumetric properties.

Components	Unit	Asphalt Mixtures		
		HMA	HMAJ	CRAJ
Grading Composition				
Limestone 31.5–10 mm	%	9	9	11
Limestone 6–12 mm	%	32	32	7
Limestone 3–6 mm	%	31	31	–
Limestone sand	%	24	24	2
Limestone filler	%	4	–	–
JGW filler	%	–	4	4
RAP	%	–	–	76
Binders by Total Weight of the Aggregates				
Bitumen	%	3.8	4.30	–
Bitumen emulsion	%	–	–	5
Cement	%	–	–	0.5
Water	%	–	–	5
Mastic				
f/b ratio	–	1.05	0.93	1.14
Volumetric Properties				
Bulk density	g/cm^3^	2.52	2.51	2.52
Air void content	%	4	4	9

**Table 6:** ITSM test loading parameters.

Test Parameters	Unit	Value
Loading pulse rise-time (see “c” in Figure 12)	ms	120
Rest period (see “d” in Figure 12)	ms	3000
Pulse repetition period	ms	3000
N° conditioning pulses	–	10
Target temperature	°C	10
Estimated Poisson’s ratio	–	0.35
Target horizontal deformation	μs	47

**Table 7:** ITSM values of HMA, HMAJ, and CRAJ, and variation Δ of HMAJ and CRAJ in comparison with HMA.

Asphalt Mixtures	ITSM at 10 °C (MPa)	Δ (%)	ITSM at 25 °C (MPa)	Δ (%)	ITSM at 40 °C (MPa)	Δ (%)
HMA	15,500	–	7350	–	3960	–
HMAJ	16,300	5	8730	18.7	4500	13.6
CRAJ	16,820	8.5	9420	28	5610	41.6

## Data Availability

The data presented in this study are available on request from the corresponding author.
